# The gut-liver axis: microbial mechanisms and therapeutic implications in MAFLD

**DOI:** 10.3389/fcimb.2026.1740163

**Published:** 2026-08-10

**Authors:** Lihong Xue, Haojie Wang, Bowen Shan, Changwen Li, Hongyan Chen, Changqing Yu, Changyou Xia, He Zhang

**Affiliations:** 1 State Key Laboratory of Animal Disease Control and Prevention, Harbin Veterinary Research Institute, Chinese Academy of Agricultural Sciences, Harbin, China; 2 School of Advanced Agricultural Sciences, Yibin Vocational and Technical College, Yibin, China

**Keywords:** gut-liver axis, metabolic dysfunction-associated fatty liver disease (MAFLD), microbial metabolites, precision medicine, traditional Chinese medicine (TCM)

## Abstract

Metabolic dysfunction-associated fatty liver disease (MAFLD) has become the most common chronic liver disease worldwide, currently affecting approximately 32% of the global adult population.Initially presenting as simple hepatic steatosis, MAFLD often progresses to severe cardiovascular complications, imposing significant healthcare and economic burdens. Current clinical management relies primarily on lifestyle interventions, with few targeted pharmacotherapies available, highlighting the urgent need for deeper mechanistic understanding and innovative treatments. A growing body of research highlights the critical role of intestinal homeostasis in the development and progression of MAFLD. Modern studies refer to the bidirectional communication between the gut and liver via the biliary tract, portal vein, and systemic circulation as the “gut-liver axis.” This structural connection makes the liver more susceptible to damage from gut-derived microbes, metabolites, endotoxins, and inflammatory mediators, positioning the liver as a key target organ exposed to the intestinal microenvironment. For instance: LPS (endotoxin) a component of Gram-negative bacteria, activates the TLR4 signaling pathway, triggering NF-κB and promoting the release of pro-inflammatory cytokines such as TNF-α and IL-6. This amplifies inflammation and compromises the intestinal barrier, allowing bacteria and their products to enter the liver via the portal vein and induce liver injury. Short-chain fatty acids (SCFAs, e.g., butyrate), produced by the fermentation of dietary fiber, inhibit histone deacetylase (HDAC) and activate G-protein-coupled receptors (e.g., GPR43, GPR109A). These mechanisms promote the differentiation of regulatory T cells (Treg) and the production of IgA antibodies, thereby suppressing inflammation and strengthening the mucosal barrier.Secondary bile acids (e.g., 3-oxoLCA), generated through microbial modification of bile acids, can regulate the Treg/Th17 balance via nuclear receptors such as the vitamin D receptor (VDR). Numerous studies have observed microbial dysbiosis in MAFLD, characterized by an increase in Bacteroidetes and a decrease in Firmicutes.Emerging therapies targeting gut microbiota—including microbial metabolite modulators (HDCA, 4-HPAA) and multi-target traditional Chinese medicines (silymarin, Cornus officinalis glycosides)—demonstrate promising therapeutic potential when combined with interventions like probiotics and fecal microbiota transplantation.

## 1 Introduction

Metabolic dysfunction-associated fatty liver disease (MAFLD) is a chronic liver disease caused by the interaction of multiple factors such as genetics and environment. It is defined as the presence of hepatic steatosis confirmed by imaging, blood tests, or liver biopsy, along with at least one of the following metabolic abnormalities: 1) overweight or obesity (BMI ≥ 25 or ≥ 23 for Asians); 2) diagnosed type 2 diabetes; 3) meeting at least two indicators of metabolic dysfunction syndrome (including abdominal obesity, hypertension, hypertriglyceridemia, low HDL cholesterol, abnormal fasting blood glucose, or insulin resistance) (Friedman et al., 2018; Eslam et al., 2020). Previously, the condition was referred to as non-alcoholic fatty liver disease (NAFLD), with core diagnostic criteria requiring imaging or histological confirmation of hepatic steatosis exceeding 5%, while excluding significant alcohol consumption (<30 g/day for men, <20 g/day for women) and other liver diseases (such as viral hepatitis). This nomenclature emphasized the non-alcoholic nature of the disease but failed to highlight its underlying metabolic abnormalities. Such naming could easily lead to the misconception that NAFLD is merely a simple accumulation of liver fat, overlooking the associated metabolic dysregulation.The term MAFLD, first and foremost, more accurately reflects the core characteristic of the disease—metabolic dysfunction. In clinical practice, the new naming simplifies the diagnostic process by no longer requiring strict exclusion of alcohol consumption and allowing for co-diagnosis with other liver diseases, thereby better aligning with real-world clinical scenarios (Fouad et al., 2020). Additionally, this naming helps reduce the stigma associated with the term “non-alcoholic, “ improving public awareness and understanding of the disease (Pipitone et al., 2023). In 2019, Eslam and other experts published a consensus explicitly recommending the renaming to MAFLD, which received 70% expert support through the Delphi method. Currently, MAFLD has been adopted by multiple international and national hepatology associations (Eslam et al., 2020; Gofton et al., 2023).

The progression of MAFLD involves multiple dynamic stages. It begins with the accumulation of lipids within the liver (simple steatosis), which is often asymptomatic. Subsequently, lipid overload triggers mitochondrial dysfunction, oxidative stress, and endoplasmic reticulum stress, leading to hepatocyte injury and inflammation, and progressing to metabolic-associated steatohepatitis (MASH). Lipotoxic metabolites (such as diacylglycerol and ceramides) further damage hepatocytes and promote inflammation and hepatic stellate cell activation, driving the development of fibrosis. Ultimately, progressive fibrosis can result in cirrhosis, portal hypertension, and a significantly increased risk of hepatocellular carcinoma (Yang et al., 2019; Chen et al., 2020; Wei et al., 2024). MAFLD has become the most common chronic liver disease worldwide, currently affecting approximately 32% of the global adult population. Research shows that there are significant gender differences in the disease, with a prevalence rate of about 40% in men and 26% in women. Since 2016, the prevalence of MAFLD has continued to rise, especially in regions such as Southeast Asia and the Americas, where it has exceeded 40% (Teng et al., 2023). Influenced by factors such as obesity rates, genetic susceptibility, and socioeconomic status, the health burden of MAFLD varies significantly among different populations. Patients not only bear high direct medical costs but also suffer from huge indirect economic burdens due to decreased productivity, chronic fatigue, pain, and psychological stress (Shao and Fan, 2019).

Emerging evidence underscores the gut-liver axis as a critical modulator of MAFLD pathogenesis. The intestinal epithelium not only facilitates nutrient/vitamin absorption and bile excretion but also mediates bidirectional communication with the liver through the portal circulation. This anatomical connection enables hepatic sensing of gut-derived metabolites and nutrients, triggering adaptive metabolic reprogramming (Khan et al., 2021). First conceptualized by Marshall (1998), the gut-liver axis encompasses multidirectional crosstalk via biliary, portal, and systemic circulation routes (Zeuzem, 2000). This physiological linkage renders the liver particularly susceptible to gut-derived insults including: (1) microbial dysbiosis, (2) pathogenic metabolites (e.g., endotoxins), and (3) proinflammatory mediators (Tilg et al., 2022). Consequently, the intestinal microenvironment directly influences hepatic metabolic and inflammatory states, establishing gut microbiota modulation as a promising therapeutic strategy for MAFLD. This review will critically evaluate mechanistic links between gut microbial communities and MAFLD progression, highlighting potential intervention targets along the gut-liver axis.

## 2 Pathogenesis of nonalcoholic fatty liver disease: a multifactorial cascade

The pathogenesis of MAFLD is not yet fully understood. The classic “two-hit hypothesis” (Day and James, 1998) posits that hepatic lipid accumulation (“the first hit”) makes the liver more susceptible to subsequent damage through oxidative stress and inflammation (“the second hit”) (Day and James, 1998), However, modern research supports a more complex “multiple-hit” model, which involves the synergistic effects of metabolic, genetic, and environmental factors (Buzzetti et al., 2016).

The core mechanism of MAFLD is insulin resistance, which disrupts lipid homeostasis, promotes lipogenesis and inhibits fatty acid β-oxidation, leading to hepatic steatosis (Ipsen et al., 2018). Lipid overload exacerbates insulin resistance and triggers oxidative stress and excessive reactive oxygen species (ROS) generation through mitochondrial dysfunction. ROS induce hepatocyte injury and activate pro-inflammatory and pro-fibrotic pathways.

Inflammatory responses further aggravate liver damage. Activated Kupffer cells and macrophages release damage-associated molecular patterns (DAMPs) and cytokines, forming a self-sustaining inflammatory cycle that promotes steatosis and fibrosis (Mohammad and Thiemermann, 2020). Autophagy plays a dual role in MAFLD: insulin resistance inhibits autophagy and increases early lipid accumulation; while in the late stage of the disease, autophagy dysregulation may accelerate hepatocyte apoptosis as it shares signaling pathways with cell death programs (Guo et al., 2022).

**Figure 1:**
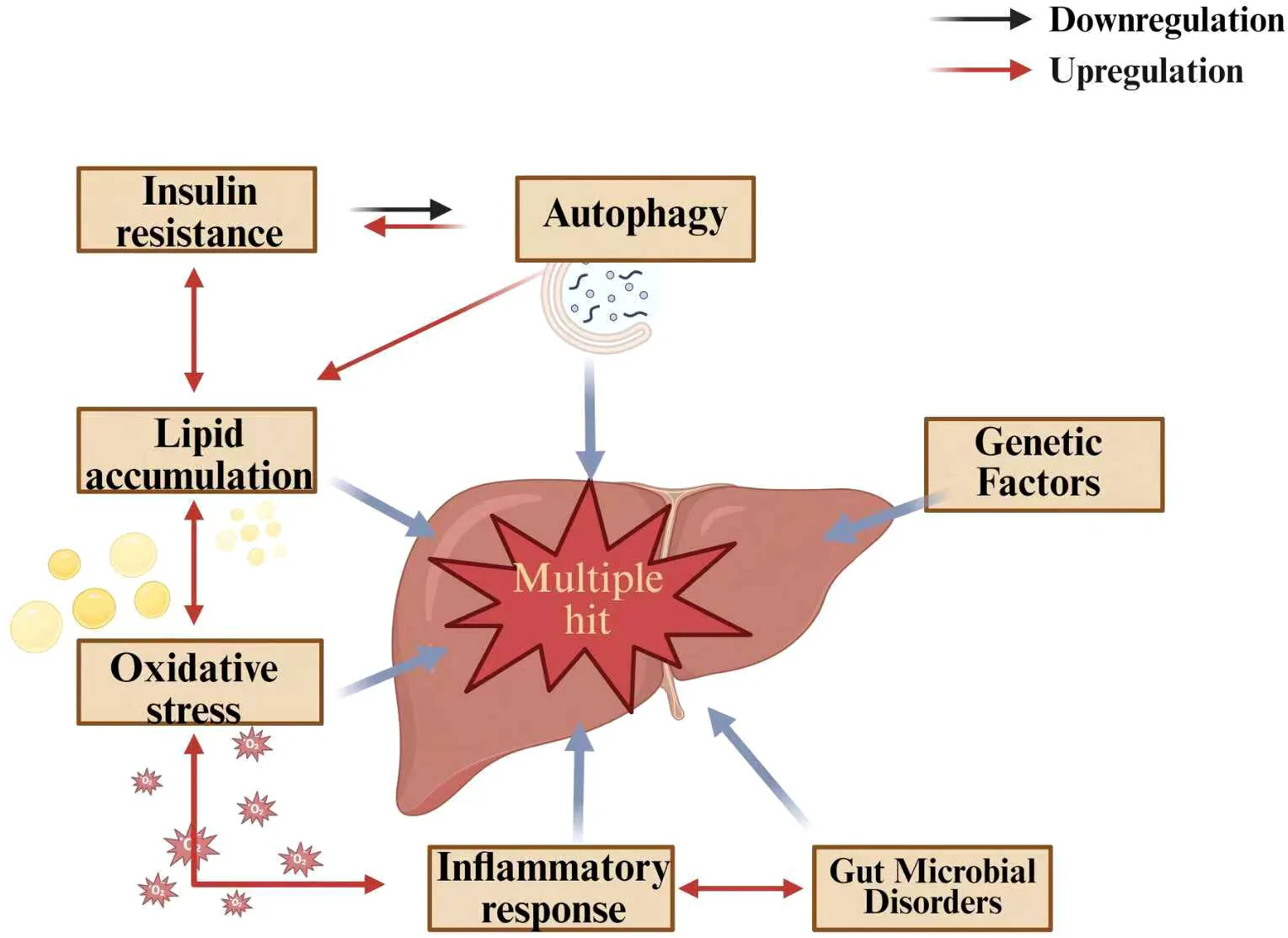
MAFLD pathogenesis.

Emerging pathogenic factors encompass a delicate interplay of intestinal microbiota imbalance, which elevates circulating endotoxin levels and reshapes metabolite landscapes, alongside genetic variations such as PNPLA3-I148M. This remarkable genetic variant orchestrates lipid metabolism and inflammatory responses, casting a profound shadow over susceptibility to non-alcoholic fatty liver disease, with carriers facing an alarming 12-fold heightened risk of hepatocellular carcinoma (Buzzetti et al., 2016). Collectively, these revelations illuminate non-alcoholic fatty liver disease as a complex tapestry woven from metabolic derangements, oxidative insults, chronic inflammation, and extrahepatic influences, underscoring its multifaceted nature (**Figure 1**).

The pathogenesis of MAFLD involves the activation and cross-regulation of multiple signaling pathways, affecting lipid and glucose metabolism in hepatocytes and regulating liver inflammation and fibrosis through molecular networks. Among them, Toll-like receptors (TLRs), nuclear receptor families (PPARs, FXR, LXR), and insulin receptors (IR) play key roles. The interaction between these receptors and related signaling pathways constitutes the molecular basis of MAFLD and provides potential targets for treatment (Bessone et al., 2019). Current therapeutic research highlights the crucial roles of the farnesoid X receptor (FXR) and peroxisome proliferator-activated receptors (PPARs). FXR is widely expressed in the liver, intestine, and kidneys, regulating lipid/cholesterol metabolism, inflammatory responses, and the composition of the gut microbiota. Bile acids (BAs), as endogenous ligands of FXR, play a dual role in MAFLD: maintaining metabolic homeostasis at physiological levels, but causing hepatotoxicity when overaccumulated, including lipid deposition, oxidative stress, inflammatory activation, and insulin resistance. Additionally, bile acid overload due to cholestasis activates hepatic stellate cells, promotes fibrosis, and disrupts the intestinal microbiota balance (Fuchs and Trauner, 2022).

The molecular mechanisms of FXR action involve sophisticated regulatory networks. BA-bound FXR forms heterodimers with RXRα, initiating three major regulatory pathways: (1) Through the FGF15/19 endocrine axis (species-specific), ileal FXR activation induces FGF15 (rodents) or FGF19 (humans) secretion, which upon hepatic FGFR4/β-Klotho receptor binding, suppresses CYP7A1 expression - the rate-limiting enzyme in BA synthesis. (2) FXR-activated small heterodimer partner (SHP) inhibits CYP7A1 transcription by binding and inactivating HNF4α and LRH-1 transcription factors. (3) FXR coordinately regulates BA transport via intestinal upregulation of IBABP and OSTα/β (enhancing enterocyte BA efflux) while downregulating ASBT (reducing luminal BA uptake), and hepatic modulation of NTCP and OATP1B1/1B3 transporters (controlling hepatocyte BA uptake/secretion). These integrated mechanisms maintain BA homeostasis, alleviate cholestatic injury, and potentially mitigate MAFLD progression (Katafuchi and Makishima, 2022).

PPARs serve as master regulators of lipid and glucose metabolism while significantly influencing energy homeostasis, inflammatory responses, and fibrotic processes (Wagner and Wagner, 2020). The PPAR family consists of three functionally distinct subtypes with differential tissue expression patterns. Hepatic-enriched PPAR-α governs lipid catabolism by transcriptional regulation of genes involved in triglyceride hydrolysis, fatty acid transport, and β-oxidation pathways (Korbecki et al., 2019). Pharmacological PPAR-α activation reduces plasma triglycerides through dual mechanisms: suppressing hepatic synthesis while enhancing lipoprotein lipase-mediated clearance. Experimental models demonstrate that PPAR-α deficiency exacerbates high-fat diet-induced hyperlipidemia and proinflammatory states. However, sustained PPAR-α overactivation carries potential adverse effects including: (1) excessive fatty acid oxidation leading to mitochondrial dysfunction and oxidative stress, (2) hepatocyte damage promoting fibrogenesis, and (3) impaired insulin signaling exacerbating metabolic dysfunction. PPAR-β/δ, predominantly expressed in skeletal muscle, regulates mitochondrial fatty acid β-oxidation and energy metabolism. Its activation ameliorates ectopic lipid accumulation in metabolic disorders, while genetic deletion elevates VLDL production and reduces lipoprotein lipase activity, thereby aggravating hepatic steatosis. PPAR-γ primarily mediates adipocyte differentiation and lipid storage while modulating insulin sensitivity, inflammatory responses, and fibrotic pathways through integrated effects on oxidative/ER stress signaling (Chen et al., 2023).

## 3 Research progress on the influence of gut microbiota on MAFLD via the gut-liver axis

### 3.1 Regulation of intestinal flora on MAFLD

The gut microbiota, often referred to as the human body’s “second genome”, is a complex ecosystem comprising bacteria, fungi, archaea, and viruses. Firmicutes dominate this community (~80%), followed by Bacteroidetes, Actinobacteria, Proteobacteria, and minor populations of Lactobacillus, Streptococcus, and Escherichia coli. Functioning as a metabolic organ, the gut microbiota plays a crucial role in maintaining host homeostasis by aiding food digestion, modulating immune responses, and generating bioactive metabolites. Its composition is shaped by genetic, environmental, and lifestyle factors, including age, diet, and medication use (Marchesi et al., 2016).

Under healthy conditions, a symbiotic equilibrium exists between the gut microbiota and the host, with the intestinal barrier effectively preventing pathogenic bacteria and their harmful byproducts from reaching the liver. However, in MAFLD, this balance is disrupted. Dysbiosis reduces the expression of tight junction proteins, compromising intestinal barrier integrity. As a result, bacteria and their metabolites—such as lipopolysaccharides (LPS), peptidoglycans, short-chain fatty acids (SCFAs), indole derivatives, and secondary bile acids—translocate across the damaged barrier, entering the liver via the portal vein. These microbial products interact with liver cells, causing metabolic disorders like hyperlipidemia, obesity, and type 2 diabetes (Canfora et al., 2019; Aron-Wisnewsky et al., 2020; Fang et al., 2022).

### 3.2 The derived components of the intestinal microbiota and their metabolites’ impact on MAFLD

Recent studies emphasize the critical role of gut microbiota in MAFLD progression. MAFLD patients often show intestinal flora dysbiosis and altered microbial metabolite profiles. The liver, sensitive to intestinal bioactive substances via the portal circulation, regulates glucose, lipid metabolism, immune responses, and redox balance in response. Identifying key gut-liver interaction pathways may offer new MAFLD therapeutic targets (Ji et al., 2019).

LPS, a major component of Gram-negative bacterial cell walls, is consistently elevated in both MAFLD patients and animal models. This increase promotes chronic low-grade inflammation, a key driver of MAFLD pathogenesis. Mechanistically, LPS activates Toll-like receptor 4 (TLR4), triggering the upregulation and secretion of multiple pro-inflammatory cytokines and chemokines. These mediators not only exacerbate oxidative stress but also directly contribute to hepatocellular injury (Vallianou et al., 2021).

The inflammatory cascade is further amplified through LPS-binding protein (LBP), which facilitates LPS transfer to CD14 for presentation to the TLR4/MD-2 complex. This process enhances LPS sensitivity, creating a self-perpetuating cycle of hepatic inflammation and damage. Notably, intervention studies using Gram-negative-targeted antibiotics have shown reduced TNF-α production and plasma LPS levels, effectively attenuating oxidative stress and inflammatory responses in the liver (Meng et al., 2022).

Beyond their direct metabolic actions, these microbial metabolites also exert profound immunomodulatory effects within the hepatic microenvironment. Importantly, the metabolic effects of SCFAs and bile acids act on hepatic macrophages and Kupffer cells via receptor activation or epigenetic reprogramming, thereby modulating the local inflammatory milieu. Such immuno-metabolic crosstalk underscores the gut-liver axis as a bidirectional rheostat that couples microbial metabolite production to both hepatic metabolic fitness and inflammatory tone, further perpetuating MAFLD pathogenesis.

SCFAs, produced through gut microbial fermentation of dietary fiber (Tan et al., 2014; Ratajczak et al., 2019). These microbial metabolites primarily function through two complementary mechanisms: activation of G-protein coupled receptors (GPR41, GPR43, GPR109a, and OLFR78) and inhibition of histone deacetylases (HDACs). Notably, propionate and butyrate demonstrate particularly potent mtabolic effects via GPR43 activation, which enhances energy expenditure while suppressing fat accumulation in liver and adipose tissue, thereby maintaining energy homeostasis (Fu et al., 2021). Beyond their metabolic roles, SCFAs exhibit robust anti-inflammatory properties, chiefly through suppression of NF-κB signaling pathway activation. Butyrate offers additional metabolic benefits by improving insulin sensitivity through GLP-1R activation, inhibiting hepatic *de novo* lipogenesis, and enhancing intestinal barrier function through tight junction protein induction. In healthy individuals, a balanced gut microbiota produces sufficient SCFAs to maintain systemic metabolic equilibrium (Silva et al., 2020). However, MAFLD-associated dysbiosis leads to reduced SCFA production, creating a pathological milieu that exacerbates both metabolic dysfunction and inflammatory responses, thereby accelerating disease progression.

BAs, the end products of cholesterol metabolism, undergo extensive microbial modification in the intestinal tract, where they acquire enhanced biological activity (Di Ciaula et al., 2017). These modified BAs exert their metabolic effects primarily through simultaneous activation of two key receptors: the FXR and Takeda G protein-coupled receptor 5 (TGR5). Through these dual pathways, BAs improve insulin sensitivity while suppressing inflammatory signaling cascades.

FXR activation modulates hepatic glucose production by altering expression of gluconeogenic genes, resulting in reduced blood glucose levels and inhibition of NF-κB-mediated inflammatory pathways (Jia et al., 2024; Zhang et al., 2024). In the distal ileum, FXR activation stimulates fibroblast growth factor 15/19 (FGF15/19) production, creating a negative feedback loop that suppresses bile acid synthesis and alleviates cholestasis (Shi et al., 2020). Concurrently, BAs enhance energy expenditure through their actions on brown adipose tissue (BAT), where they boost mitochondrial activity and upregulate thermogenic gene expression. This process drives non-shivering thermogenesis, contributing to both body temperature regulation and systemic energy balance (Castellanos-Jankiewicz et al., 2021).

Emerging research highlights additional benefits of BA signaling through TGR5 activation, including promotion of intestinal epithelial cell regeneration and improvement of gut barrier integrity (Fuchs and Trauner, 2022). Importantly, microbial transformation of primary BAs into secondary forms enhances their potency in metabolic and immune regulation (Sorrentino et al., 2020). These secondary BAs demonstrate superior efficacy in modulating both metabolic pathways and immune responses compared to their primary precursors.

**Figure 2:**
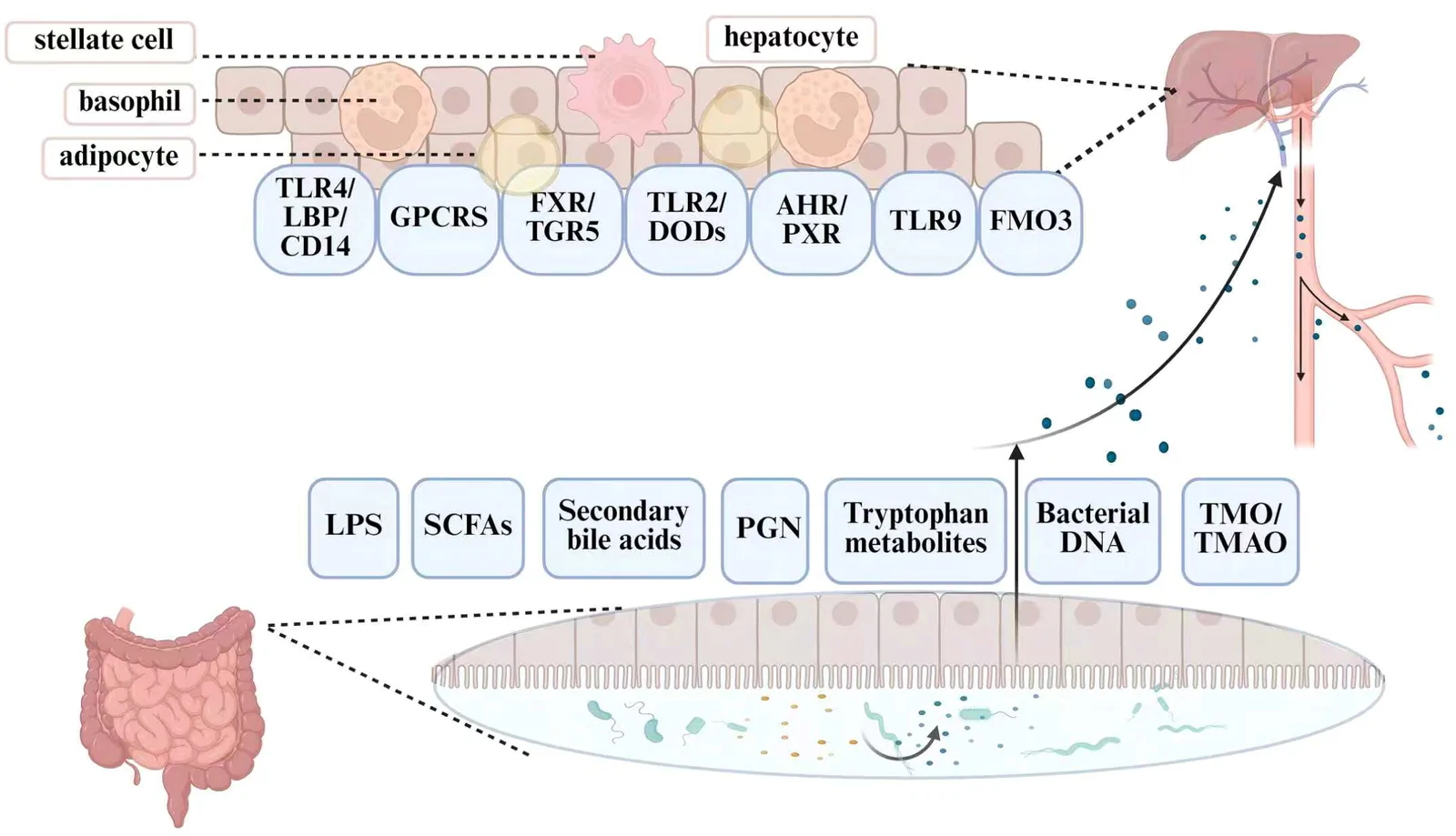
The mechanisms that connect gut microbiome-derived ingredients and metabolites with MAFLD. MAFLD induces profound alterations in the gut-liver axis. Beneficial commensal bacteria are replaced by potentially pathogenic species, leading to dysbiosis and bacterial overgrowth. Increased penetrability of the mucus layer and enhanced permeability of the epithelial and vascular barriers facilitate the translocation of bacteria and their products. Translocated microbial metabolites act on target cells including hepatocytes, Kupffer cells, hepatic stellate cells, and adipocytes through distinct receptor systems: LPS/TLR4/NF-κB drives pro-inflammatory cytokine production; SCFAs/GPR43/HDAC promotes immune regulation and barrier protection; secondary bile acids/FXR/TGR5 modulates BA homeostasis, energy metabolism, and inflammatory tone. Other microbial factors (PGN, EVs, indole derivatives, TMAO, and CpG DNA) engage distinct receptor systems (TLR2, NOD1/2, AhR, PXR, TLR9) to collectively contribute to hepatic steatosis, inflammation, and fibrosis. LPS, lipopolysaccharide; SCFAs, short-chain fatty acids; HDAC, histone deacetylase; FXR, farnesoid X receptor; TGR5, Takeda G protein-coupled receptor 5; PGN, peptidoglycan; EVs, extracellular vesicles; AhR, aryl hydrocarbon receptor; PXR, pregnane X receptor; TMAO, trimethylamine N-oxide.

Beyond bile acids and SCFAs, MAFLD development involves multiple additional microbial-derived factors including peptidoglycan (PGN), extracellular vesicles (EVs), tryptophan metabolites, trimethylamine derivatives, carotenoids, and bacterial DNA. Each contributes uniquely to disease progression through distinct mechanisms: PGN, a major component of Gram-positive bacterial cell walls, activates pattern recognition receptors (TLR2, NOD1, and NOD2) in hepatic tissue, triggering pro-inflammatory cytokine release (Jin et al., 2020). High-fat diet-induced dysbiosis typically increases Firmicutes populations, elevating circulating PGN levels that compromise intestinal barrier integrity and amplify hepatic inflammation through immune cell activation. Microbial-derived EVs represent another important mediator, functioning as nanoscale carriers of bioactive molecules. While certain EV components can paradoxically strengthen intestinal barrier function, most activate hepatic TLR and NOD receptors to perpetuate inflammatory cascades (Sun et al., 2022). Tryptophan metabolites, particularly indole derivatives, exert protective effects through aryl hydrocarbon receptor (AhR) and pregnane X receptor (PXR) activation. These compounds demonstrate multi-faceted benefits including: enhanced insulin sensitivity, reduced hepatic steatosis, strengthened intestinal barriers, Treg cell induction, and attenuated inflammation (Ala, 2022; Wang and Zeng, 2023). The gut microbial metabolite trimethylamine (TMA) and its oxidized product TMAO have emerged as potential early biomarkers for metabolic syndrome. Meanwhile, bacterially modified carotenoids and phenolic compounds contribute to MAFLD protection through their antioxidant properties and glucose metabolism regulation (Rath et al., 2019; Konopelski and Mogilnicka, 2022; Yan et al., 2025). Finally, bacterial DNA promotes hepatic inflammation through TLR9 activation, which is particularly elevated in NASH patients and facilitates inflammatory cell chemotaxis (Białkowska et al., 2023). This pathway completes a complex network where diverse microbial products collectively influence MAFLD progression through metabolic, inflammatory, and immunological mechanisms (**Figure 2**).

## 4 Drugs to treat MAFLD by gut microbiota

The gut microbiota plays a critical role in MAFLD progression through the gut-liver axis, with microbial-derived metabolites serving as key regulators of metabolic and inflammatory pathways. This has led to the identification of several promising therapeutic strategies. Among them, BA signaling has emerged as a major target, with drugs such as FXR agonists (e.g., obeticholic acid) and TGR5 agonists demonstrating potential to improve insulin sensitivity and suppress hepatic inflammation. Similarly, SCFAs exert beneficial effects by activating GPR43 and GPR109a, which modulate immune responses and metabolic homeostasis. Pharmacological agents that mimic these SCFA-mediated pathways could offer novel treatments for MAFLD by reducing inflammation and improving glucose and lipid metabolism (Hong et al., 2021; Liu et al., 2023).

Beyond receptor-targeted therapies, interventions that directly modulate the gut microbiota—including probiotics, prebiotics, and selective antibiotics—have shown efficacy in preclinical models. These approaches aim to restore microbial balance, enhance barrier function, and reduce the production of harmful metabolites. Additionally, microbial metabolite-based therapies, such as indole derivatives that activate AhR and PXR, are being explored for their anti-inflammatory and metabolic benefits (Chen and Vitetta, 2020).

Current research highlights the potential of gut microbiota-targeted drugs, with several candidates demonstrating promising results in animal studies (**Table 1**). Future therapeutic strategies may combine receptor-specific drugs with microbiome-modulating interventions to simultaneously address metabolic dysfunction, inflammation, and gut barrier integrity in MAFLD.

Recent research has identified HDCA as a promising therapeutic bile acid for MAFLD, with clinical studies showing disease severity correlates with HDCA depletion. In murine models, HDCA administration significantly improves key MAFLD parameters including hepatic steatosis, inflammation, and metabolic markers (TC, TG, ALT, AST). The therapeutic mechanism involves a sophisticated gut-liver axis interplay rather than direct hepatocyte action (Hindson, 2023).

HDCA operates through three interconnected mechanisms: First, it modulates intestinal FXR signaling, suppressing FGF15/19 production and subsequently activating both CYP7A1 and CYP7B1 enzymes. This shifts bile acid synthesis from the classical CYP7A1-dominated pathway to the alternative CYP7B1-mediated route, alleviating cholestasis (Katafuchi and Makishima, 2022). Second, HDCA induces beneficial gut microbiota changes, particularly enriching Prevotella species within the Bacteroidetes phylum. Metabolomics analysis reveals that microbial changes increase γ-linolenic acid and other fatty acid metabolites in the liver and cecum. Elevated γ-linolenic acid activates hepatic PPARα, forming a feedback loop that stimulates FXR and inhibits CYP7A1, promoting metabolic shifts. HDCA has clear advantages over current single-target therapies. This compound can reshape metabolic pathways in both hosts and microorganisms, underscoring the potential of gut-liver axis-targeted therapy for non-alcoholic fatty liver disease (Chen et al., 2025).

New research indicates that 4-hydroxyphenylacetic acid (4-HPAA), a gut microbiota metabolite derived from dietary polyphenols, may be a potential candidate for treating MAFLD. By implanting a 4-HPAA sustained-release device into diet-induced obese mice, researchers found that it significantly improved hepatic steatosis and reduced triglyceride accumulation. These changes were accompanied by the regulation of the liver’s gene expression profile, particularly affecting fatty acid metabolism and inflammation-related pathways. Mechanism research shows that 4-HPAA exerts beneficial effects by activating the AMPK signaling pathway. Phosphoproteomics analysis indicates that 4-HPAA significantly enhances the phosphorylation of AMPKα and its downstream target ACC, promoting fatty acid oxidation and inhibiting lipogenesis. This dual metabolic effect was further verified in a dose-response study of primary mouse hepatocytes (Wang et al., 2018).

A bioactive component, theaflavin (TB), found in tea has been proven to effectively improve MAFLD and alleviate obesity by regulating the gut-liver axis. Research indicates that TB exerts its therapeutic effect by differentially regulating serotonin metabolism in various tissues. Metabolomic analysis shows that TB significantly alters the tryptophan metabolic pathway, reducing serotonin levels in the liver while increasing concentrations in serum and visceral adipose tissue (Suchacki et al., 2023). The mechanism of action of TB involves multiple coordinated effects: it down-regulates the expression of HTR2A in the liver while up-regulating the expression of PPARα and CYP4A14 to enhance fatty acid oxidation; it activates HTR2B in adipose tissue, promoting the phosphorylation of HSL and fat breakdown. Additionally, TB reduces serotonin degradation by inhibiting the expression of SERT and MAO-A, thereby alleviating oxidative stress. Crucially, these effects rely on the participation of the gut microbiota - antibiotic treatment eliminates the benefits of TB, while fecal microbiota transplantation can restore its efficacy, indicating that TB requires a complete microbiota to regulate host serotonin homeostasis (Li HY. et al., 2023; Zhen et al., 2023).

The polysaccharide α-D-1, 3-glucan (RPP-2), derived from Radix Puerariae thomsonii, demonstrates significant therapeutic potential for metabolic disorders through its multi-faceted mechanisms of action. As a potent prebiotic, RPP-2 selectively enhances the proliferation of beneficial Lactobacillus plantarum strains P9 and JZ6, establishing a foundation for its systemic metabolic effects. In high-fat diet models, RPP-2 administration effectively ameliorates glucose intolerance and improves hepatic pathology, as evidenced by significant reductions in key metabolic markers including hepatic total cholesterol, triglycerides, and serum aminotransferase levels (Li Q. et al., 2023).

The metabolic benefits of RPP-2 emerge through an integrated series of molecular events. By modulating gut microbiota composition, RPP-2 alters bile acid metabolism, particularly increasing secondary bile acid production. These microbial-derived bile acids then activate the farnesoid X receptor, initiating a cascade of metabolic regulation. Concurrently, RPP-2 influences the PPAR signaling pathway, potentially activating PPARα, PPARδ and PPARγ isoforms to coordinately regulate lipid homeostasis (Clifford et al., 2021; Qiu et al., 2023). This dual modulation results in decreased lipogenesis and enhanced fatty acid oxidation, while simultaneously improving inflammatory responses.

These findings position RPP-2 as a promising therapeutic candidate that uniquely bridges microbial ecology and host metabolism. Its ability to simultaneously target gut microbiota composition, bile acid metabolism, and PPAR-mediated signaling pathways suggests potential applications for MAFLD and related metabolic disorders, offering a holistic approach to metabolic regulation through the gut-liver axis. The compound’s prebiotic properties combined with its systemic metabolic effects highlight the therapeutic potential of microbiota-targeted interventions for metabolic diseases.

**Table 1:** Drugs targeting gut microbiota for MAFLD treatment in preclinical mouse models.

Drugs	Bioactive species	Treatment mechanism	Key gut flora	References
HDCA	Bile acids	Inhibition of intestinal FXR activates the alternative CYP7B1-centered pathway and inhibits the classical CYP7A1-centered pathway via Paraboidus-G-linolenic acid-Ppara signaling, thereby altering the hepatic BA synthesis pathway	Parabacteroides distasonis	(Kuang et al., 2023)
TB	Tea polyphenols	Regulation of serotonin levels in the liver by the gut microbiota	Akkermansia, Bacteroides, Parabacteroides	(Li HY. et al., 2023)
PRR-2	Plant polysaccharides	It regulates BA anabolism to activate FXR and activates PPARα	Lactobacillus, Butyricicoccus, Oscillibacter	(Li Q. et al., 2023)
4-HPAA	Dietary polyphenols	After being metabolized by intestinal microorganisms, AMPK signaling pathway is activated	Parabacteroides distasonis	(Osborn et al., 2022)
Resveratrol	Natural polyphenols	It alleviates hepatic steatosis and insulin resistance by modulating gut microbiota, restoring intestinal barrier function, improving metabolic pathways, and reducing inflammation.	Olsenella, , Oscillibacter, Clostridium_IV , Bacteroides, Streptococcus, Enterorhabdus	(Du et al., 2021)
Berberine/baicalin	Alkaloids/flavonoids	Intestinal flora increases BA content and activates FXR-FGF15/FGFR4 pathway to exert negative feedback regulation of BA	Bacteroides, Parabacteroides	(Gao, 2024)
Silymarin	Flavonoids	Increase the abundance of bacteria with higher vitamin B12 synthesis capacity and reduce lipid accumulation	Akkermansia muciniphila, Escherichia/Shigella, Bilophila wadsworthia	(Sun et al., 2023)
Diosgenin	Tetracyclic triterpenoids	Activation of the FXR-SHP and FXR-FGF15 pathways reduces BA synthesis	Bacteroides, Clostridium	(Yan et al., 2023)

## 5 Summary and outlook

In recent years, with the continuous increase in the incidence of MAFLD, it has become a significant public health problem worldwide. The severe multi-system complications induced by MAFLD, such as cardiovascular diseases, have imposed a huge burden on healthcare. Currently, clinical management mainly relies on lifestyle changes, and there are few drugs that can achieve long-lasting therapeutic effects. There is an urgent need for a deeper understanding of the pathogenesis of MAFLD and the development of innovative treatment approaches for MAFLD.

The complexity of the pathogenesis of MAFLD has made the traditional “two-hit” hypothesis no longer applicable. Instead, a more comprehensive “multi-hit” theory has emerged. This theoretical framework particularly emphasizes the synergistic role of immune dysfunction, genetic susceptibility, impaired autophagy, and intestinal microbiota dysbiosis in the progression of MAFLD. Through the gut-liver axis, intestinal microbial metabolites - including short-chain fatty acids, bile acids, lipopolysaccharides, peptidoglycans, and tryptophan derivatives - coordinate key metabolic and inflammatory processes in the development of MAFLD.

Targeting the gut microbiota has yielded promising therapeutic candidates:

HDCA and 4-HPAA modulate bile acid metabolism and AMPK signaling; RPP-2 acts as a prebiotic to enhance fatty acid oxidation; TB differentially regulates serotonin metabolism across tissues; Traditional Chinese medicines (e.g., silymarin, Cornus officinalis glycosides) exhibit multi-target effects through microbiota remodeling.

These agents demonstrate efficacy in improving lipid metabolism, reducing inflammation, and restoring gut barrier function. Particularly noteworthy is the unique advantage of Traditional Chinese Medicine (TCM), which combines microbiota modulation with pleiotropic regulation of oxidative stress, insulin resistance, and metabolic pathways. Clinical applications of TCM compounds like Salvia miltiorrhiza and Astragalus membranaceus, alongside microbiota-targeted interventions (probiotics, fecal microbiota transplantation), have shown significant promise.

Future directions should focus on: Mechanistic Elucidation: Deeper investigation into microbial-host crosstalk in MAFLD progression; Precision Medicine: Integration of metabolomics and genomics for personalized treatment strategies; Therapeutic Innovation: Development of multi-target TCM formulations and synthetic microbial consortia; Standardization: Addressing challenges in TCM quality control and treatment protocols.

While challenges remain in addressing individual variability and therapeutic standardization, the convergence of microbiota science, multi-omics technologies, and TCM principles offers unprecedented opportunities for MAFLD treatment. By leveraging these interdisciplinary approaches, the next generation of therapies may finally provide effective, tailored solutions for this multifactorial disease.
